# Probable Importation of Dengue Virus Type 4 to Angola from Brazil

**DOI:** 10.3201/eid2010.140609

**Published:** 2014-10

**Authors:** Eyal Meltzer, Yaniv Lustig, Ora Glichinsky, Florian Steiner, Eli Schwartz

**Affiliations:** The Chaim Sheba Medical Center Department of Medicine 'C' and Center for Geographic Medicine, Tel Hashomer, Israel (E. Meltzer, E. Schwartz);; Tel Aviv University Sackler School of Medicine, Tel Aviv, Israel (E. Meltzer, E. Schwartz);; Ministry of Health Central Virology Laboratory, Tel Hashomer (Y. Lustig, O. Glichinsky;; Institute of Tropical Medicine and International Health, Charité-Universitätsmedizin Berlin, Berlin, Germany (F. Steiner)

**Keywords:** dengue fever, dengue virus, Africa, Angola, travel, viruses, importation, transmission

**To the Editor:** The prevalence of dengue virus (DENV) infection in Africa may have been grossly underestimated for many years (*1*). Even though published reports are scarce, dengue has been documented in at least 34 countries in Africa (*2*). The role of travelers as sentinels of infectious disease has proven to be invaluable in this regard; dengue in returning travelers was the only evidence for local DENV transmission for 12 of these countries.

During April 2013, simultaneous reports of travelers returning from Luanda, Angola, with dengue fever emerged from Israel, several countries in Europe, Canada, and South Africa; PCR and sequencing of viral RNA confirmed the causative species to be DENV type 1 (DENV-1) (*3*). Concomitantly, a large outbreak of dengue was confirmed by local authorities in Luanda, and DENV-1 was isolated in samples from local residents (*4*). The origin of the outbreak strain was not ascertained, but phylogenetic analysis suggested that it had close affinity with isolates from West Africa (*4*,*5*). These reports involved almost 100 travelers and >500 residents of Luanda who become within a short time. Active DENV transmission and occurrence of new cases in Luanda were still ongoing during early July 2013 (*6*), but since then, to our knowledge, no additional local data on DENV activity in Luanda have been published.

We report 3 new cases of dengue fever acquired in Luanda during December 2013–February 2014. Two cases occurred in travelers who returned to Israel, and 1 occurred in a traveler who returned to Germany. All 3 cases occurred in middle-aged businessmen who had traveled to Angola and who were hospitalized within days of returning to their home countries because of signs and symptoms of dengue fever. None of the case-patients met criteria for severe dengue, and all recovered uneventfully. Acute DENV infection was confirmed by serologic testing in all 3 patients and by positive results for a nonstructural protein 1 serum antigen test in 2 travelers.

In a serum sample taken from 1 of the travelers from Israel on the second day of fever, DENV RNA was detected by using reverse transcription PCR. This strain was found to belong to DENV type 4 (DENV-4). Phylogenetic analysis was performed, and multiple sequence alignment of this DENV-4 sequence, in comparison to other DENV-4 sequences retrieved from GenBank, was performed by using the Sequencher 5.0 program (Gencodes Corporation, Ann Arbor, MI, USA). A maximum-likelihood phylogenetic tree was inferred from the sequence alignment by using ClustalX (http://www.clustal.org), and the robustness of the tree was assessed by 1,000 bootstrap replications. The tree was visualized and produced by using NJ plot software (http://doua.prabi.fr/software/njplot). Results showed that RNA from this isolate was most closely related to that of a DENV-4 strain identified in 2010 in Boa Vista, the capital of Roraima State in the Amazon Region of Brazil (*7*) (Figure).

**Figure:**
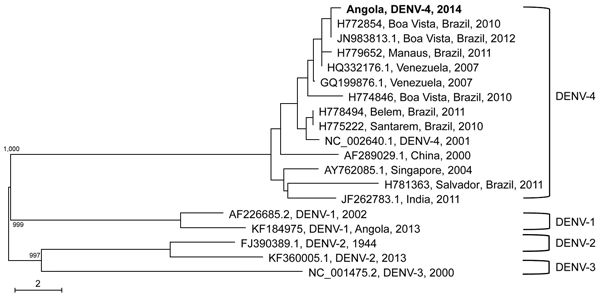
Phylogenetic analysis of a dengue virus (DENV) type 4 strain isolate (boldface) from a patient in Israel who apparently acquired DENV in Angola in 2014, showing close relationship with isolates from Brazil. The DENV isolate was aligned with representative DENV sequences from around the world, representing serotypes 1–4. Reference strains, downloaded from public databases (http://www.ncbi.nlm.nih.gov/nuccore), are identified by accession number, place, and year of isolation (DENV-4 isolates) or by serotype, accession number, and year of isolation (DENV-1–3 isolates). Specific branches indicate bootstrap values. Scale bar indicates percentage identity difference.

DENV-4 infection was previously reported in Africa in 1986, when 2 cases were identified in travelers returning from Senegal (*8*), but to our knowledge, no other cases have been reported in western or southwestern Africa. During the April 2013 dengue outbreak in Luanda, only DENV-1 was isolated (*3**,**4*). After that outbreak, a study conducted on the basis of modeling of international commercial flight data to and from Angola suggested that DENV would most likely have been imported from Latin America (*5*). Our finding, a year later, of DENV-4 in Angola that was closely related to strains from Brazil appears to vindicate this modeling system. During the past decade, Brazil had been experiencing a consistent increase in dengue epidemics and in severity of disease (*9*). Brazil’s large and growing economy, its increasingly prominent place in world trade, and its growing tourism industry highlight the country’s potential role in the global circulation of DENV.

Our findings are corroborated by a recently reported case of dengue in a traveler from Portugal that was acquired in Luanda concomitantly with our cases and also found to be caused by DENV-4 (*10*). In light of the apparent introduction of DENV-4 to Angola, probably from Brazil, health authorities should be encouraged to enhance surveillance and vector control efforts. In addition, health practitioners treating travelers returning from Angola should be aware of the risk for DENV infection.
